# A systematic review of self-medication practice during the COVID-19 pandemic: implications for pharmacy practice in supporting public health measures

**DOI:** 10.3389/fpubh.2023.1184882

**Published:** 2023-06-15

**Authors:** Yu Zheng, Jiayu Liu, Pou Kuan Tang, Hao Hu, Carolina Oi Lam Ung

**Affiliations:** ^1^State Key Laboratory of Quality Research in Chinese Medicine, Institute of Chinese Medical Sciences, University of Macau, Taipa, Macao SAR, China; ^2^Department of Public Health and Medicinal Administration, Faculty of Health Sciences, University of Macau, Taipa, Macao SAR, China

**Keywords:** self-medication, pharmacist, COVID-19, public health, systematic review

## Abstract

**Introduction:**

Since the COVID-19 pandemic, self-medication had become highly popular due to the risk of virus infection and overwhelming medical resources. Pharmacists are well-positioned to provide public health education and disease prevention. This study aims to provide an overview of the research about self-medication during COVID-19 and the role of pharmacists in ensuring the drug safety related to self-medication.

**Methods:**

Databases (PubMed, Google Scholar, Scopus, EBSCO host, and Web of Science) were searched for published studies on the practice of self-medication in COVID-19 pandemic without restriction in population and location. Primary search terms were “self-medication,” “self-care,” “self-management,” “non-prescription drugs,” “2019nCoV,” and “COVID-19.” Studies conducted during the pandemic but not exclusively for COVID-19 disease were eligible for inclusion.

**Results:**

The database search yielded a total of 4,752 papers. After appropriate screening, 62 articles met the inclusion criteria. Most of the studies were cross-sectional in nature. The review highlighted a very high prevalence of self-medication during COVID-19, ranging from 7.14 to 88.3%. The purpose of self-medication was mainly to treat and prevent COVID-19; fever, body aches, cough, headache, and sore throat were the most frequently mentioned indications. Categories of drugs commonly used in self-medication included antibiotics, herbs, vitamins, and analgesics, most of which came from pharmacies. Information about self-medication usually obtained from relatives and friends, social networks and health care professionals. Common reasons for self-medication included saving money and time, prior experience and mild illness; reasons associated with COVID-19 were mainly fear of contracting the virus and poor access to doctors. Gender, age, education, marital status, and concern about COVID-19 were the most usual associated factors. The role of pharmacists in self-medication included sources of information, advice on medication use, and management of adverse reactions.

**Conclusion:**

During the COVID-19 pandemic, self-medication practices were widespread and varied across countries and populations. Self-medication has emerged as an important component of health care, but also as a huge global challenge. The engagement of healthcare administrators and policy makers are essential to regulate self-medication practices. The expertise and favorable conditions of pharmacists make them positioned as key roles in public health interventions for self-medication.

**Systematic review registration:**

https://www.crd.york.ac.uk/prospero/display_record.php?RecordID=395423, identifier CRD42023395423.

## 1. Introduction

Self-medication is defined by WHO as treatment of self-recognized disorders or symptoms by use of medicines without prior consultation by a qualified health professional or intermittent/continued use of medicines previously prescribed by a physician for chronic/recurring diseases (1). Self-medication is a widespread habit throughout the world and is considered an essential part of health policy in various countrie (2–7). This is especially evident during a pandemic such as the COVID-19 infection. Makowska et al. showed that a number of people experienced their first involvement in self-medication during the pandemic (8). A study in Kenya also found that the total prevalence of self-medication among health care workers increased to 60.4% during the COVID-19 pandemic from 36.2% before the pandemic (9). In Pakistan, the rate of self-medication behavior among medical students during the pandemic was as high as 83% (10). Self-medication behavior was also prevalent among the general public in India (59.9%) (11).

Since the COVID-19 pandemic, self-medication had become highly popular due to a number of reasons. Initially, due to the known risk of contracting the virus, people are afraid to go to clinics or hospitals (12). Also, there are challenges in accessing medical settings due to lockdown and restriction policies (12). Furthermore, COVID-19 may make the issue of poor access to healthcare even worse, particularly in nations with underdeveloped health systems (13). With the unexpected patient burden and inadequate healthcare human resources resulting from healthcare worker infections, healthcare services may be hampered (14). Quite a few governments have also urged people to self-medicate for minor symptoms to avoid crowding out medical resources. All of these may have contributed to people opting for self-medication.

Self-medication, when properly used, can benefit both individuals and health systems in a number of ways, including reducing the amount of time spent waiting in line for medical appointments, preventing limited medical resources from being used on minor conditions, reducing the workload of doctors, lowering health care costs, and lowering absenteeism from work (15, 16). Regardless of the unquestionable benefits obtained from self-medication practice, there are undesired outcomes that may result from improper usage. These have been mentioned in studies where self-medication may have involved risks of misdiagnosis, administration of an excessive dose, improper duration of use, and adverse drug reactions associated with improper medication use (17, 18). Inappropriate self-medication may lead to irrational use of drugs, waste of resources, increase in polypharmacy, and interactions with other frequently used drugs and delays in treatment (19). Additionally, antibiotic overuse fuels the emergence of drug-resistant pathogens worldwide (3).

Pharmacists play a crucial role in recognizing, resolving, and avoiding drug-related problems in order to achieve the best possible patient outcomes and quality of life (15). They are professionally trained to support and assist patients in making informed health decisions (20). Considering that the products used for self-medication is mostly accessed through the pharmacy (21), pharmacists are well-positioned to deliver public health education and disease prevention. Notably, pharmacist involvement in the use of over-the-counter (non-prescription) medications is widely recognized and has the potential to improve patient outcomes (22). The International Pharmaceutical Federation (FIP) report “Pharmacy as a gateway to care: Helping people toward better health” emphasizes the idea of facilitated or advised self-medication as well as the role that pharmacists can play as facilitators to the self-care decisions consumers take in the selection and use of over-the-counter (non-prescription) medicines (23).

Nevertheless, little has been reported about the interface between self-medication during the COVID-19 pandemic and the role of pharmacists. Previous studies have explored the use of self-medication for COVID-19 disease (21, 24, 25). However, a broader systematic review is necessary to integrate all self-medication behaviors during the pandemic to provide better insight into public health in this resource-constrained setting. Also, there is a lack of research on the role of pharmacists in self-medication during the pandemic, and understanding this situation would contribute to the promotion of the pharmacists' role. Therefore, the objective of this review was to provide an overview of the research about self-medication during COVID-19 and the role of pharmacists in ensuring the drug safety related to self-medication. The overall goal is to promote responsible self-medication, thus making contributions to public health in future pandemics.

## 2. Methods

This systematic review was conducted and reported in accordance with the Preferred Reporting Items for Systematic Reviews and Meta-Analyses (PRISMA) guidelines (26) (Supplementary material 1). The study protocol has been registered on the PROSPERO systematic review database (CRD42023395423).

### 2.1. Search strategy

Online digital libraries were used to search for relevant papers on July 22 2022. We systematically searched the following electronic databases: PubMed, Scopus, EBSCO host, Web of Science and gray literature sources (Google Scholar). Search terms were derived from two main keywords: “self-medication,” and “COVID-19.” Primary search terms were “self-medication,” “self-care,” “self-management,” “self-treatment,” “non-prescription drugs,” “otc drugs,” “drug utilization,” “2019nCoV,” and “COVID-19.” The specific search equations utilized for each database are shown in Supplementary material 2. Additionally, bibliographic citations of included studies were reviewed to identify other relevant studies.

### 2.2. Eligibility criteria

All original articles published in English concerning self-medication practices during COVID-19 were reviewed. Studies conducted during the pandemic but not exclusively for COVID-19 disease were eligible for inclusion. The selection of articles did not include any population and location restrictions. Self-medication was defined as taking medicines to treat health conditions or symptoms without prescription or diagnosis from a qualified healthcare professional (9). It might involve a range of production including, but not limited to, over-the-counter drugs, antibiotics, traditional and complementary medicine (including herbal product and dietary supplements). If a study provided a definition of self-medication in the article or if the medication addressed in the article did not require a medical prescription, we regarded that study as having investigated about self-medication. Case reports, book, comment, letter, reviews, news, preprint article and editorials were excluded.

### 2.3. Study selection

The PRISMA Flow Diagram was used to select the articles for this review. Results of our searches were imported EndNote, where duplicates were removed. Two reviewers, YZ and JY, independently screened studies based on eligibility criteria. They manually reviewed the databases' search results first by title and abstract in accordance with the inclusion criteria. After that, they examined the full text of the relevant papers to decide whether they were suitable for inclusion in the study. Any divergences between the two reviewers were discussed. Should reviewers not reach accord, a third reviewer CU participated in the process to make a final decision on inclusion or exclusion.

### 2.4. Data extraction

Utilizing a Microsoft Excel spreadsheet, two authors (ZY and JY) independently extracted the following details for the review: author, year of publication, study area, study population, study design, sample size, sampling technique, date of data collection, how was self-medication assessed, prevalence of self-medication, types of self-medication, condition for self-medication, reasons to practice self-medication, and factors associated with self-medication, sources of drugs, sources of self-medication information, adverse event, knowledge as well as attitudes associated with self-medication.

## 3. Results

### 3.1. Study selection

**Figure 1:**
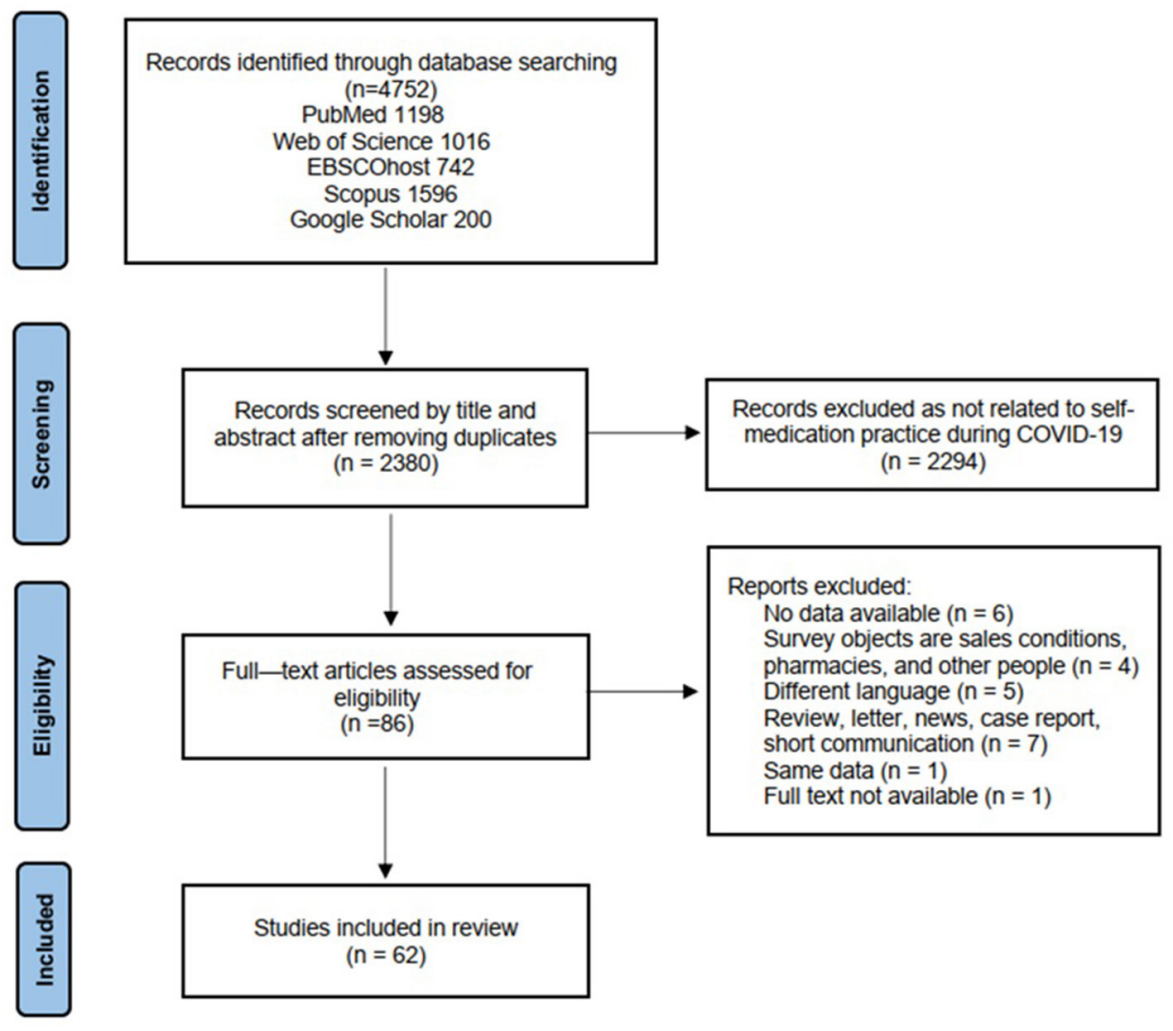
PRISMA flowchart showing selection strategy.

In total, 4,752 articles were kept through our preliminary database search. When the duplicate articles were adjusted, there were 2,380 articles left. After screening the titles and abstracts of the remaining 2,380 studies, 2,294 records were excluded since they didn't match the criteria for inclusion. We retrieved and reviewed the full text of 86 articles. As a result, 62 articles met the inclusion criteria. The remaining 24 papers were removed (five beyond the required language, seven article types out of scope, six studies without available data, one article full text is not available, one article had the same data, and four articles were not targeted at the participants themselves; Figure 1).

#### 3.2. Study characteristics

##### 3.2.1. Basic information of the included studies

The 62 included studies reported about the practice of on self-medication during COVID-19 in 29 different countries, of which 35 studies were from the Asian region namely Iran (*n* = 5) (12, 27–30), China (31), Indonesia (*n* = 2) (32, 33), Thailand (34), India (*n* = 8) (11, 35–41), Pakistan (*n* = 5) (10, 42–45), Turkey (*n* = 3) (46–48), Bangladesh (*n* = 5) (49–53), Saudi Arabia (*n* = 3) (54–56), Jordan (57), Nepal (58); seven from Europe continent, Romania (59), Poland (*n* = 2) (8, 60), Netherlands (*n* = 4) (61–64), Ireland (62), Norway (*n* = 3) (62–64), Switzerland (*n* = 2) (62, 64), UK (*n* = 2) (62, 64), Sweden (63), Belgium (64); four from South America, Peru (*n* = 3) (65–67), Colombia (68); three from North America, Mexico (*n* = 3) (69–71); one from Oceania, Australia (72); and 12 from Africa, Kenya (*n* = 2) (9, 73), Togo (*n* = 2) (74, 75), Ethiopia (76), Nigeria (*n* = 6) (77–82), and Sub-Saharan Africa (83).

Regarding the population, 30 studies were conducted targeting the general population (8, 11, 30, 31, 34–36, 38, 40, 42, 44, 47, 49–51, 54–57, 61, 63, 65, 67, 68, 70, 72, 75, 79, 81, 83), 10 was conducted in students (10, 33, 39, 41, 43, 53, 58, 60, 71, 78), four in dental visit (12, 29, 45, 46), three in healthcare workers (9, 80, 82), three in individuals visited medical stores and medical store owners (73, 76, 77), two in older adult (28, 48), two in patient (27, 66), one in COVID-19 recovered patients (52), one in mothers with school-age children (32), one in pregnant and breastfeeding women (62), one in pregnant and postpartum women (64), one in adults with a history of taking allopathic medication in the last month (37), one in five doctors with different specialties (interviews) and adults (survey) (59), one in people with symptoms related to anxiety and depression (69), and one in workers from five sectors (health care, air transport, police, road transport, and informal) (74).

The vast majority of the selected studies used a cross-sectional survey design and 1 used mixed-method (i.e., cross-sectional surveys with qualitative work) (59). All studies together included 75,262 participants with sample sizes ranging from 80 to 16,724. The majority of the study were conducted in 2020 and 12 in 2021. Studies were performed from January 2020 to December 2021.The study characteristics collected from the reviewed literature are fully described in Table 1.

**Table 1:** Characteristics of the included studies.

**No**.	**References**	**Country**	**Study design**	**Sample population**	**Sample size**	**Collection period**	**Purpose of self-medication**	**Prevalence of self-medication %**	**Type of product used for self-medication**
1	Khami et al. (12)	Iran	CS	Dental visits	756	mid-4 2020 to mid-7 2020	Dental problem	Before pandemic 26.5% After pandemic 56.1%	Amoxicillin 22.7%, incomplete information 14.8%, azithromycin 4.7%, co-amoxiclav 3.8%, amoxicillin and metronidazole 3.5%, metronidazole 2.3%, penicillin 1.7%, cefixime 0.9%, doxycycline 0.9%, clindamycin 0.6%, and azithromycin and penicillin 0.3%
2	Lam et al. (31)	China	CS	General people	632	2020.11.2–2020.12.18	Prevention of COVID-19 and others	All 54.1% Before pandemic 48.4% During pandemic 44%	Vitamins or other dietary supplements 25.3%, Chinese herbal medicine 19.3%, and Western herbal medicine 5.1%
3	d'Arqom et al. (32)	Indonesia	CS	Mothers with school-age children	610	2020.7–2020.12	Prevention and/or treatment of COVID-19	NR	Medication: Antibiotics 42%, antivirus 16%, antimalaria 5%, and others 37%; Vitamins: Vitamin C 39%, multivitamins and minerals 32%, vitamin E 11%, vitamin D 8%, Zinc 6%, and Selenium 1%; Herbs/Natural product: Ginger 31%, honey 30%, curcumin 22%, eucalyptus 5%, and other 12%.
4	Goodwin et al. (34)	Thailand	CS	General people	1,000	2020.4.20–2020.5.3	Prevention of COVID-19	15.0%	Vitamins or other medicines
5	Onchonga et al. (9)	Kenya	CS	Healthcare workers	379	NR	Treatment for specific symptoms	Before pandemic 36.1% After pandemic 60.4%	NR
6	Mahmoudi (27)	Iran	CS	COVID-19 patients	436	2020.3.11–2020.10.13	Treatment of COVID-19	56.1%	Antibiotics
7	Sarkar and Rajamani (35)	India	CS	General people	200	2020.8–2020.11	Treatment for specific symptoms	65%	Diclofenac and paracetamol
8	Arain et al. (42)	Pakistan	CS	General people	698	2020.4–2020.9	Treatment for specific symptoms	NR	OTC, antibiotics, and others (sedatives)
9	Sadio et al. (74)	Togo	CS	The healthcare, air transport, police, road transport and informal sectors	955	2020.4.23–2020.5.8	Prevention of COVID-19	34.2%	Vitamin C 27.6%, traditional medicine 10.2%, chloroquine/hydroxychloroquine 2.0%, and azithromycin 1.2%.
10	Oktarlina et al. (33)	Indonesia	CS	Medical faculty students	252	2020.1.1–2020.1.10	Treatment for specific symptoms	NR	Supplement and drugs
11	Coman et al. (59)	Romania	Mixed method (interview and survey)	Interviews: five doctors with different specialties. Survey: predominantly adults and student	543	2021.1–2021.4	Prevention and treatment of COVID-19	NR	Symptomatic medicines for allergies, respiratory, gastric, pain, anxiolytic antibiotics, vitamins, anti-thermics, oral disinfectants, antispasmodics, anti-diarrheals, and non-steroidal anti-inflammatory medication (ibuprofen, diclofenac, etc.)
12	Tossou (75)	Togo	CS	Households	1,946	2020.7.8–2020.7.17	No specific indication	61.41%	NR
13	Tandon et al. (36)	India	CS	General people	312	NR	Prevention of COVID-19 and others	NR	Prophylactic therapy with the perception to prevent COVID-19 infection (*n* = 4), VC (*n* = 2), hydroxychloroquine (*n* = 1).
14	Mansuri et al. (54)	Saudi Arabia	CS	General people under lockdown	388	2020.3–2020.4	Prevention and treatment of COVID-19	Self-medication for fever 35.1%	NR
15	Sen Tunc et al. (46)	Turkey	CS	Parents who applied to dental clinic regarding their children's dental problems	389	2020.7–2020.10	Dental problem	70.2% (self-medicated their children)	Analgesics 98%, antibiotics 38.1%, mouthwashes 13.1%, and herbal medicines 8.8%
16	Sikdar et al. (49)	Bangladesh	CS	General people	2,941	2020.11.25–2020.12.4	Sleep disturbances	7.14%	NR
17	Tekeba et al. (76)	Ethiopia	CS	Community-pharmacy clients	416	2020.6.1–2020.6.30	Treatment for specific symptoms	73.6%	Painkillers 83.7%, antibiotics 10.5%, cough syrup 1.6%, antacid 1.6%, oral contraceptive 1.3%, and other 1.3%
18	Soriano-Moreno et al. (65)	Peru	CS	General people	3,610	2020.9.7–2020.9.21	Prevention and treatment of COVID-19	Prevention: 8% Treatment: 16.4%	Chlorine dioxide
19	Merwid-Lad et al. (60)	Poland	CS	The students during the academic year 2020/2021	624	2021.11.14–2021.12.23	Anxiety, depression, or sleeping problems and others	70%	Dietary supplements: Magnesium or a combination of magnesium with vitamin B6, *Melissa officinalis* L. (melissa, lemon balm), melatonin, a Vitamin-B group complex, *Valeriana officinalis* L. root (valerian root), *Matricaria recutita* L. (wild chamomile), *Withania somnifera* L. (ashwagandha), *Humulus lupulus* L. (hop), cannabidiol (CBD) oil, ginseng, vitamin D with or without menaquinone-7 (MK7), vitamin C, vitamin B12, multivitamin preparations, zinc, iron, and omega-3 fatty acids OTC: Antihistamines, analgesic, antipyretic, and anti-inflammatory drugs
20	Yusuf and Sarkinfada (77)	Nigeria	CS	Individuals that visited medical stores and medical store owners	332	NR	Treatment for specific symptoms	During pandemic 68.5% Before pandemic 64.2%	Artemisinin combination therapy 39.5%, Co-trimoxazole 16.0%, amoxicillin 14.1%, ciprofloxacin 12.9%, ampicillin-cloxacillin 6.7%, tetracycline 6.7%, and chloroquine 3.7%
21	Bello et al. (78)	Nigeria	CS	Nigerian undergraduates	356	2020.5–2020.8	Prevention and treatment of COVID-19	65.4%	Vitamin C 52%, paracetamol/panadol 43%, herbs 28.7%, anti-malaria 24.7%, food supplements 15.7%, cough syrup 8.1%, slimming pills and teas 6.5%, piriton 3.4%, anti-diarrhea 2.2%, tramadol 2%, hydroxychloroquine 2%, and other 15.2%
22	Gupta and Chakraborty (37)	India	CS	Adults with a history of taking allopathic medication in the last month	170	2020.8	Treatment for specific symptoms	57.7%	Pain suppressor 46.9%, antibiotics 43.9%, anti-acidity 39.8%, and anti-allergics (including cough suppressants) 16.3%
23	Akintunde et al. (83)	Sub-Saharan Africa	CS	General people	536	2020.8.18–2020.8.24	No specific indication	38.8%	NR
24	Saleem et al. (43)	Pakistan	CS	Undergraduate students	520	2020.3–2020.6	Treatment for specific symptoms	58.1%	Analgesics 55.96%, antibiotic 38.74%, antipyretic 34.44%, antihistamine 20.86%, vitamins 17.55%, antiemetic 9.27%, antidiarrheal 8.28%, antacids 5.96%, laxatives 4.97%, food supplements 4.30%, and others 13.58%
25	Tobaiqi et al. (55)	Saudi Arabia	CS	General people	281	2020.7–2020.9	Treatment for specific symptoms	58%	Analgesics 42.9%, antibiotic 14.1%, herbs 13.5%, vitamins 9.2%, eye drops 6.1%, antacid 4.9%, roquia treatment 2.5%, laxatives 0.6%, and other 6.1%
26	Heshmatifar et al. (28)	Iran	CS	Older adult>60	342	2020	Prevention of COVID-19	56.4%	Pain reliever 52%, vitamins and supplements 47%, anti-cold 44%, sedative 42.6%, antibiotics 27.1%, gastrointestinal drugs 25.9%, and cardiac drugs 17%
27	Mulder et al. (61)	Netherlands	CS	General people	1,004	2020.5.22–2020.5.27	Prevention and treatment of COVID-19 and others	59.4%	Homeopathic remedies 10.2%, Bach flowers 4.1%, and Herbal medicine 19.0%: Echinacea, Passiflora, curcumin, red yeast rice (Xuezhikang), milk thistle (Silybum marianum), ashwagandha (Withania somnifera), cranberry, black cohosh, ginseng, and ginkgo biloba Vitamins/minerals 55.0%: Multivitamins, vitamin C, vitamin D, vitamin B, selenium, zinc, iron, magnesium, and calcium Other CM 14.0%: Omega 3, 6, 9, co-enzyme Q10, protein drink/shake, probiotics, and glucosamine-chondroitin-MSM
28	Elayeh et al. (57)	Jordan	CS	General people	1,179	2020.3.26–2020.4.16.	Prevention and treatment of COVID-19	80.4%	Antibiotics: azithromycin and doxycycline; Analgesics and antipyretics: paracetamol, ibuprofen, and diclofenac; Minerals: zinc, magnesium, and iron salts; Vitamins: vitamins C, D, and B and multivitamins; Herbals and supplements: propolis, omega 3 fatty acids, and immune boosting supplements; Antithrombotic drugs: aspirin and enoxaparin; Cold and cough preparations; Antihistamines; Others: antiseptic lozenges, nasal solutions containing normal saline or sea water, clove oil, and menthol rub.
29	Chopra et al. (38)	India	CS	General people with middle and high socioeconomic status	1,100	2020.5.1–2020.5.10	Anxiety	25%	NSAIDS 36%, antiulcer drugs 18%, H1 Anti-histaminics, 15%, multivitamins 7%, antimicrobials 6%, herbal drugs 3%, and hydroxychloroquine 1%
30	Azhar et al. (56)	Saudi Arabia	CS	General people	290	2020	Prevention of COVID-19	53%	Allopathic medicines: Azithromycin 21.5%, cough syrup 16.7%, soften 15.6%, disprin 5.2%, ivermectin 3.3%, dexa methasone 3%, and hydroxychloroquine 2.6% Herbals: Sana makhi tea 32.6%, green tea 4.8%, homeopathic medicines 3.3%, lemon tea 2.4%, ginger tea 2%, joshanda tea 1.6%, and tootsiah syrup 0.4% Vitamins: Vitamin C 27%, surbex Z 18.9%, vitamin D 18.5%, Tab. calcium 14.8%, multi-vitamins 2%, centrum 0.4%, and folic acid 0.4%
31	Amuzie et al. (79)	Nigeria	CS	General people	469	2021.10–2021.11	Prevention and treatment of COVID-19	30.3%	Herbal products 43.7%, anti-malarials (ACTs) 34.5%, vitamin supplements 28.2%, azithromycin 23.9%, ivermectin 12.7%, analgesics 12%, calcium supplement 8.5%, hydroxychloroquine 8.5%, and ciprofloxacin 4.9%
32	Okoye et al. (80)	Nigeria	CS	Health care professionals	669	2021.3–2021.4	Prevention and treatment of COVID-19	36.3%	Ivermectin 9.5%, azithromycin 9.1%, vitamin C 7.4%, chloroquine 5.7%, and zinc sulfate 2.0%
33	Acharya et al. (58)	Nepal	CS	Medical students and staffs	383	2021.11.1–2021.11.30	Prevention and treatment of COVID-19	50.4%	Paracetamol 18.9%, vitamin C 18.6%, zinc 12.7%, multivitamins 11.1%, vitamin D 9.6%, azithromycin 8%, cough syrup 7.8%, ibuprofen 6.8%, calcium 3.2%, ivermectin 1.2%, montelukast 0.7%, dexamethasone 0.6%, chloroquine 0.3%, and other 0.4%
34	Gaviria-Mendoza et al. (68)	Colombia	CS	General people	397	2020.6.30–2020.9.14	Prevention of COVID-19 and treatment for specific symptoms	34.3%	Nervous system: Analgesics 86.0%, acetaminophen 85.3%, and other (psycholeptics and psychoanaleptics) 5.9% Musculoskeletal system: Anti-inflammatory and anti-rheumatic products 47.1%, muscle relaxants 3.7% Respiratory system: Antihistamines for systemic use 26.5%, cough and cold preparations 24.3% Alimentary tract and metabolism: Vitamins 21.3%, drugs for acid-related disorders 16.9%, and other (drugs for constipation, anti-diarrheals, etc) 6.6% Anti-infectives for systemic use: Anti-bacterials for systemic use 12.5%, anti-mycotics for systemic use 2.2% Blood and blood forming organs (antithrombotic agents) 13.2% Antiparasitic products, insecticides, and repellents: Antiprotozoals 3.7% and anthelmintics 2.9% Other: Systemic hormonal preparations 2.2%, cardiovascular system 1.5%, dermatologicals 0.7%, and other (natural products) 19.1%
35	Rafiq et al. (44)	Pakistan	CS	General people	920	2020.3–2020.8	Treatment for specific symptoms	Total 63.7% Among adults 67.3% Among teenagers 46.9%	NR
36	Vasquez-Elera et al. (66)	Peru	CS	Patients hospitalized in COVID-19 areas of the Cayetano Heredia Hospital who self-medicated before admission.	301	2020.5–2020.6	Treatment of COVID-19	54.8%	Ivermectin 85.5%, azithromycin 71.5%, corticosteroids 46.7%, and NSAIDs 31.5%
37	Wegbom et al. (81)	Nigeria	CS	General people	461	2020.6–2020.7	Prevention and treatment of COVID-19	41%	Vitamin C and multivitamin 51.8%, other antimalarial drugs 47.1%, amoxicillin 24.9%, ciprofloxacin 14.6%, herbal products 10.2%, metronidazole 8.5%, erythromycin 5.3%, and hydroxychloroquine and chloroquine 3.2%
38	Quispe-Cañari et al. (67)	Peru	CS	General people	3,792	2020.6.5–2020.6.17	Prevention and treatment of COVID-19	33.4%	Acetaminophen 27%, ibuprofen 7.4%, azithromycin 4.8%, penicillin 2.3%, antiretrovirals 1.6%, and hydroxychloroquine 0.7%
39	Yasmin et al. (10)	Pakistan	CS	Medical Students	489	2021.1.25–2021.2.20	Prevention and treatment of COVID-19	83%	Paracetamol 65.2%, multivitamins 56.0%, ibuprofen 29.0%, cetirizine 27.8%, azithromycin 25.6%, hydroxychloroquine 8.8%, antivirals 7.2%, ivermectin 4.5%, doxycycline 3.9%, and others 11.4%
40	Zhang et al. (72)	Australia	CS	General people	2,217	2020.3.16–2020.4.1	Prevention of COVID-19	19.5%	Antibiotics
41	Makowska et al. (8)	Poland	CS	General people	1,013	2020.6.8–2020.6.15	Prevention of COVID-19 and others	45.6%	NR
42	Ceulemans et al. (62)	Ireland, Norway, Switzerland, The Netherlands, and United Kingdom (UK)	CS	Pregnant and Breastfeeding Women	7,260	2020.6.16–2020.7.14	No specific indication	Pregnant women: 22.0% Breastfeeding women: 16%	Medications, folic acid, multivitamins, iron-containing preparations, omega-3 fatty acids, and other products (including but not limited to pre- and probiotics, herbal remedies and homeopathic products)
43	Alonso-Castro et al. (69)	Mexico	CS	Population with symptoms associated with anxiety and depression	2,100	2020.3–2020.6	Anxiety and depression	61.9%	Orange blossom (*n* = 524), chamomile (*n* = 508), valerian (*n* = 419), tilia (*n* = 360), passion flower (*n* = 353), cinnamon (*n* = 171), ginkgo (*n* = 153), toronjil (*n* = 134), hierba de San Juan (*n* = 110), aloysia citrodora (*n* = 90), and marijuana (n=44)
44	Karataş et al. (47)	Turkey	CS	General people	389	2020.4.1–2020.4.30	Prevention of COVID-19	39.3%	Herbal medicines 30.8% and nutritional supplements/vitamins 23.8%
45	Ruiz-Padilla et al. (70)	Mexico	CS	General people	16,724	2020.3–2020.11	Prevention of COVID-19	35.3%	Acetaminophen, aspirin, ibuprofen, dexamethasone, hydroxychloroquine, chloroquine, azithromycin. ivermectin, chlorine dioxide, transfer factor, green tea, zinc, vitamin C, lemon, curcuma, ginger, propolis, and ginseng
46	Ahmed et al. (50)	Bangladesh	CS	General people	1,222	2020.6.27–2020.7.20	Prevention of COVID-19 and treatment for specific symptoms	NR	Allopathic medicines 15%: Arsenicum album 30.4%, vitamin supplements (vitamin C, D, B, and multivitamins) 27.1%, mineral supplements (mostly zinc) 19.9%, paracetamol 16.0%, antihista, mines (fexofenadine, desloratadine, and chlorpheniramine) 11.6%, antiasthmatics (mostly montelukast) 8.8%, and ivermectin 5.5%, Herbal 56.7%: Tea (normal and herbal) 70.9%, ginger 56.5%, black seed 32.8%, honey 30%, clove 28.8%, cinnamon 23.0%, garlic 16.9%, lemon 13.6%, black pepper 8.8%, cardamom 2.8%, bay leaf 2.1%, and tulsi 1.8%
47	Kristoffersen et al. (63)	Norway, Sweden and the Netherlands	CS	General people	2,494	2020.4–2020.6	Prevention or treatment of COVID-19 and treatment of COVID-19 related symptoms	62.8%	Herbs 18.2%: Ginger 6.8%, curcumin 5.8%, garlic 4.2%, green tea 4.0%, herbal tea 4.0%, cranberry 3.9%, blueberry/blueberry extract 3.5%, oregano 2.6%, echinacea 2.1%, aloe vera 2.0%, ginseng 1.8%, red yeast rice (xuezhikang) 1.8%, rhodiola rosea 1.0%, passiflora 0.7%, ginkgo biloba 0.6%, Indian ginseng 0.5%, actaea racemosa (black cohosh) 0.5%, chaga 0.3%, lady's thistle 0.2%, and Other herbs 3.1% Vitamins and minerals 49.9%: Vitamin D 21.2%, Multivitamins 17.5%, Vitamin C 15.7%, Magnesium 11.4%, Vitamin B 7.8%, Calcium 4.7%, Iron 4.2%, Zinc 2.7%, Selenium 1.1%, and Other vitamines and minerals 5.3% Homeopathic remedies 4.7% Bach flower remedies 2.3% Dietary supplements 29.2%: Omega 3, 6, or 9 including cod liver oil 22.2%, Protein shake 4.5%, Probiotic 3.1%, Glucosamine 1.9%, Q10 0.8%, and Other dietary supplements 2.5%
48	Mutua et al. (73)	Kenya	CS	The pharmacy customers and the pharmacy workers	80	2020.6–2020.7	Treatment for specific symptoms	78%	Anti-pyre-tics, NSAIDS, antibiotics, sedatives and hypnotics, nutritional supplements, and herbal/traditional medicines
49	Farooq et al. (39)	India	CS	Dental students and interns	100	2021.9–2021.11	Treatment for specific symptoms	34.4%	Acetaminophen 41.9%, combination of paracetamol, propyphezone & caffeine 18.3%, ibuprofen 14%, aspirin 8.6%, diclofenac 3.3%, mefenamic acid 3.2%, and ketoprofen 1.1%
50	MCPS and Malik (45)	Pakistan	CS	Dental patients	451	2020.9.20–2020.12.5	Dental problem	86.25%	Pain relievers 68.5%, antibiotics 35.5%, other 18.6%, and steroids 6.5%
51	Aitafo et al. (82)	Nigeria	CS	Health workers	220	2021.1.2–2021.3.2	Prevention and treatment of COVID-19	15.9%	Vitamin C 97.1%, zinc 80.0%, azithromycin 68.6%, anti-malarials (not hydroxychloroquine) 45.7%, hydrochloroquine/chloroquine 34.3%, multivitamins 31.4%, combination of antibiotics 14.3%, amoxicillin/clavulanic acid 8.6%, erythromycin 5.7%, amoxicillin 5.7%, ciprofloxacin 2.9%, and metronidazole/flagyl 2.9%
52	Ikiisik et al. (48)	Turkey	CS	Older adult >65	390	2021.2.22–2021.3.19	Prevention of COVID-19 and treatment for specific symptoms	48.7%	Analgesics 75%, anti-gribal 14%, antibiotics 5.7%, and vitamin 5.2%
53	Nasir et al. (51)	Bangladesh	CS	General people	626	2020.4–2020.5	Prevention of COVID-19 and treatment for specific symptoms	88.33%	Ivermectin 77.15%, azithromycin 54.15%, montelukast 43.13%, calcium supplements 41.37%, doxycycline 40.25%, hydroxychloroquine 20.44%, zinc 19.81%, and vit-d 13.58%
54	Alavi Namvar et al. (29)	Iran	CS	Dental patients	306	2020.10–2021.4	Dental problem	53.9%	Ibuprofen 23.6%, salt and water mouthwash 20.9%, amoxicillin 17.7%, acetaminophen 10.7%, metronidazole 2.9%, novafen 2.7%, mefenamic acid 1.9%, penicillin 0.3%, and others (herbs, garlic, onion, honey, lime juice, local analgesics, local salt, lidocaine, and Dentol) 19.3%
55	González-González et al. (71)	Mexico	CS	University students	284	2021.2–2021.4	Prevention of COVID-19	20.4%	Vitamins 53.9%, medicinal drugs 17.1%, herbal 10.5%, alcohol 6.6%, chlorine dioxide 3.9%, and others 7.9%
56	Chellappan et al. (40)	India	CS	General people	478	2020.9.1–2020.11.30	Prevention of COVID-19	84.5%	Home remedies 50.2%, Allopathy 46.3%: Vitamins and other dietary supplements 51.7%, medication to reduce fever 14.1%, antibiotics 12.1%, hydroxychloroquine 7.2%, painkillers 4.2%, ivermectin 3%, other medication 7%; homeopathy 32.2%, ayurveda 16.6%, naturopathy 4.5%, siddhi 3.5%, unani 0.5%, and other systems 0.2%
57	Likhar et al. (41)	India	CS	Medical students	394	NR	No specific indication	73.85%	Allopathy 43.65%: antibiotics 53.04%, anti-pyrectics 17.25%, anti-fungal 3.29%, anti-malarial 0.2%, any other 30.20%; homeopathy 8.12%, ayurvedic 7.86%, mixed 11.42%
58	Sujan et al. (52)	Bangladesh	CS	COVID-19 recovered patients	360	2020.9–2021.2	Prevention/treatment of COVID-19	11%	Paracetamol 30.6%, herbal products/drugs 30%, and antibiotics 29.7%
59	Mir et al. (11)	India	CS	General people	168	2021.5	Treatment of COVID-19-like symptoms	59.9%	Paracetamol 85.0%, azithromycin 58.0%, cough syrup 30.0%, ivermectin 18.0%, doxycycline 16.0%, ibuprofen 13.0%, dexamethasone 7.0%, hydroxychloroquine 4.0%, famotidine 3.0%, penicillins 3.0%, remdisivir 2.0%, budesonide inhalations 1.0%, and others 19.0%
60	Gerbier et al. (64)	Norway, Belgium, Switzerland, the Netherlands, and the United Kingdom	CS	Pregnant and postpartum women	5210	2021.6.10–2021.8.22	No specific indication	Pregnant women: 18.0% Postpartum women: 22.5%	Pregnant women: paracetamol 32.8%, alginic acid 5.2%, ordinary salt combinations as antacids (combinations of calcium, aluminum, and magnesium) 4.6%; Postpartum women: paracetamol 76.6%, ibuprofen 29.2%, and cetirizine 4.1%
61	Dehghan et al. (30)	Iran	CS	General people	782	2020.4.20–2020.8.20	No specific indication	84%	Medicinal herbs 48.8%: chamomile, thyme, ginger, mint, cinnamon, Imam Kazim medicine (a mixture of myrobalan, fennel, and brown sugar), hollyhocks, lavender, pennyroyal, buttercup, jujube, rosemary, viper's-buglosses, fennel, and a mixture of apple cider vinegar and honey Nutritional supplements 61.3%: vitamin D, vitamin C, multi-vitamin, and others, including vitamin B6, vitamin B complex, vitamin E, zinc, calcium, iron, omega-3, and folic acid, or a combination of supplement
62	Johora et al. (53)	Bangladesh	CS	Medical Students	916	2020.10.1–2020.10.31	Prevention of COVID-19 and treatment for specific symptoms	51.6%	Paracetamol 88.37%, anti-histamine 48.20%, vitamin C 39.96%, zinc 31.08%, ORS 20.51%, NSAIDs 20.30%, vitamin D 19.03%, vitamin E 15.01%, montelukast 14.16%, calcium 13.95%, anti-ulcerants 9.73%, sedatives 9.30%, anti-emetics 9.10%, bronchodilators 5.71%, antispasmodic 4.65%, antitussive 4.02%, herbal 3.17%, oxygen 0.63%, and others 5.50%

Abbreviations: CS, cross-sectional study; NR, not reported.

#### 3.3. Self-medication practice during COVID-19 pandemic

##### 3.3.1. Prevalence of self-medication during COVID-19

The prevalence of self-medication during COVID-19 differed across the study populations, ranging from 7.14 to 88.3% (Table 1). Six studies did not calculate overall prevalence, of which two articles investigated self-medication behavior by scale (33, 59), three articles explored only consumption/use of different medication types separately (32, 36, 50), and one article only mentioned the proportion of symptoms corresponding to product use (42).

The study with the highest prevalence (88.33%) was a public survey conducted in Bangladesh that investigated self-medication for prevention of COVID-19 and treatment of COVID-19-like symptoms (51). However, also in Bangladesh, the prevalence of self-medication for prevention or treatment of COVID-19 was only 11% among patients recovering from COVID-19 (52). The study with the lowest prevalence (7.14%) was also from Bangladesh and investigated self-treatment of sleep disorders in the general population during the pandemic (49). There are seven studies reporting the prevalence of self-medication in India, ranging from 25 to 84.5% (11, 35, 37–41). Of these, self-medication use for anxiety in general population with medium to high socioeconomic status showed the lowest prevalence rate. Two studies reporting self-medication among the student population also showed different results [34.4% (39); 73.85% (41)]. Self-medication was a common practice among several Iranian populations, including dental patients [56.1% (12); 53.9% (29)], COVID-19 patients [56.1% (27)], the older adult [56.4% (28)], and the general public [84% (30)].

In student populations, the prevalence of self-medication during the pandemic ranged from 20.4 to 83% (10, 39, 43, 53, 58, 60, 71, 78). The prevalence of self-medication among health workers in the included studies had a wide span of 15.9% (82), 36.3% (80), and 60.4% (9), respectively. In the older adult, the prevalence of self-medication was 48.7% (48) and 56.4% (28). There was a generally high prevalence of self-medication among pharmacy clients and pharmacy owners, ranging from 68.5 to 78% (73, 76, 77). Additionally, throughout the pandemic, the rate of self-medication for dental issues ranged from 53.9 to 86.25% (12, 29, 45, 46).

##### 3.3.2. Major of health conditions managed with self-medication during COVID-19

Of the included studies, 35 publications examined self-medication behavior regarding COVID-19 disease, involving prevention of COVID-19, treatment of COVID-19, and post-recovery prevention of COVID-19. Thirteen articles discussed self-medication behaviors to treat specific symptoms during the pandemic, but not limited to COVID-19 disease. Four articles considered self-medication during the COVID-19 pandemic in terms of psychological problems, another four discussed dental problems, and six articles did not clearly express the purpose of self-medication.

Altogether, 29 studies reported indications for self-medication. The most common condition was respiratory symptoms/infections, with seven studies referring to this general category. The corresponding specific symptoms included cold/flu (*n* = 7), cough (*n* = 15), runny nose (*n* = 7), nasal congestion (*n* = 3), rhinitis (*n* = 1), and sore throat (*n* = 13). Following were fever/any high body temperature (*n* = 21), body ache or joint and muscle pains (*n* = 20), headache or migraine (*n* = 14), gastrointestinal symptoms (*n* = 9) including diarrhea, vomiting, gastritis, and loss of taste and smell (*n* = 6). Other areas covered dental problems (*n* = 6), sleep problems (*n* = 4), allergy (*n* = 4), fatigue (*n* = 5), superficial wound/skin rash (*n* = 2), urinary tract infection (*n* = 1), and dysmenorrhea (*n* = 1; Table 2).

**Table 2:** Major health conditions managed with self-medication during COVID-19 pandemic.

**Condition**	**No. of studies**	**References**
Any high body temperature/fever	21	(10, 11, 33, 35, 37, 40, 42–44, 51, 53–58, 66, 67, 73, 76, 77)
Joint and muscle pains/body ache	20	(9–11, 28, 33, 38–40, 42, 43, 51, 53, 55–58, 67, 68, 73, 76)
Cough	15	(10, 11, 33, 37, 40, 42, 43, 51, 53, 55, 56, 58, 66, 67, 76)
Headache and migraine	14	(9–11, 28, 33, 38, 39, 42, 43, 53, 55, 66, 67, 76)
Sore throat	13	(10, 11, 28, 40, 43, 51, 53, 55–58, 67, 76)
Diarrhea	9	(33, 43, 44, 51, 53, 56, 58, 66, 76)
Cold/flu	7	(33, 37, 39, 40, 42–44)
Running nose/Sneezing	7	(9–11, 40, 53, 55, 67)
Respiratory infection/symptom	7	(10, 42, 66–68, 73, 77)
Loss of smell and taste/anosmia	6	(10, 51, 53, 56, 58, 66)
Dental problem	6	(12, 29, 39, 45, 46, 55)
Weakness and lethargy/fatigue	5	(10, 28, 40, 53, 67)
Allergy	4	(42–44, 76)
Nasal congestion	3	(10, 11, 67)
Sleeping problem	4	(38, 42, 53, 76)
Vomiting	4	(33, 53, 66, 76)
Superficial wound/skin rash	2	(53, 73)
Rhinitis	1	(44)
Gastritis	1	(76)
Neurological diseases	1	(28)
Cardiovascular disorders	1	(28)
Pseudo corona symptoms	1	(28)
Systemic symptoms	1	(68)
Urinary tract infection	1	(42)
Dysmenorrhea	1	(73)

##### 3.3.3. Types of medications frequently used in self-medication

The type of drug used for self-medication was reported in mostly all articles, with only eight articles failing to do so. Of the remaining studies, 18 studies didn't specify agents but instead supplied category terms, such as antibiotics, analgesics, herbal products, vitamins, and dietary supplements, in contrast to 36 studies that provided the precise names of agents, such as amoxicillin, ibuprofen, and vitamin c. A significant amount of research investigated multiple types of self-medication, while a few studies (*n* = 7) were limited to specific types of self-medication conditions, such as the use of antibiotics, painkillers, herbal medicines, and chlorine dioxide. The specific types of medications for each literature are described in detail in Table 1.

In included studies, antibiotics (*n* = 35) were the most frequently mentioned class of drugs, followed by herbs and natural products (*n* = 25), vitamins (*n* = 23), analgesics and antipyretics (*n* = 21), dietary supplements (*n* = 19), and minerals (*n* = 17; Table 3). Also common are anti-malarials (*n* = 16), antihistamines (*n* = 12), ivermectin (*n* = 12), and cough suppressants (*n* = 9). Furthermore, preferences for use varied across studies. According to the questionnaire results from different studies, painkillers like paracetamol, acetaminophen, or vitamins like vitamin C were always the most frequently used drug classes in the responses (Table 1). However, according to two research from Nigeria (79) and Turkey (47), herbal medicines were shown to be the most popular type of medication. In a similar vein, three surveys (51, 66, 80) discovered that ivermectin was the drug used for self-medication the most commonly.

**Table 3:** Categories of medicinal agents used for self-medication.

**Drug class**	**Names of specified medications in the studies**
**Prescription medicines and over-the-counter products**	
Antibiotics (*n* = 35)	Amoxicillin, azithromycin, metronidazole, penicillin, cefixime, doxycycline, clindamycin, ciprofloxacin, and erythromycin
Analgesics and Antipyretics (*n* = 21)	Diclofenac, acetaminophen/paracetamol, aspirin, ibuprofen, mefenamic acid, and ketoprofen
Antimalarial (*n* = 16)	Chloroquine, hydroxychloroquine, and artemisinin
Antihistamines (*n* = 12)	Piriton, cetirizine, fexofenadine, desloratadine, chlorpheniramine, and famotidine
Anthelmintics (*n* = 12)	Ivermectin
Cough syrup/suppressants (*n* = 9)	
Antacids (*n* = 6)	
Corticosteroids (*n* = 5)	Dexamethasone
Sedative (*n* = 4)	
Antivirus (*n* = 2)	Remdisivir
Antithrombotic (*n* = 2)	Aspirin and enoxaparin
Antiemetic (*n* = 2)	
Laxatives (*n* = 2)	
**Traditional and complementary medicine**	
Herbs and natural products (*n* = 25)	Lingzhi, Yinqiao Jiedu Pian, Radix Isatidis, Glycyrrhizae Radix Et Rhizoma, Chrysanthemi Flos, ginseng, red yeast rice, milk thistle, black cohosh, ginkgo biloba, oregano, aloe vera, rhodiola rosea, echinacea, curcumin, ashwagandha, ginger, honey, eucalyptus, cranberry, propolis, green tea, lemon, and garlic
Vitamins (*n* = 23)	Vitamin C, vitamin B, vitamin D, vitamin E, and multivitamins
Dietary supplements (*n* = 19)	Omega 3, 6 or 9 including cod liver oil, probiotics, glucosamine, melatonin, co-enzyme Q10, and protein drink/shake
Minerals (*n* = 17)	Zinc, selenium, iron, magnesium, and calcium
Homeopathic remedies (*n* = 5)	
Bach flowers remedies (*n* = 2)	
**Other**	
Chlorine dioxide (*n* = 3)	

##### 3.3.4. Major reasons for the practice of self-medication

Thirty-three of the included studies mentioned the reasons for self-medication. In relation to the pandemic, we found that the most common reasons for self-medication included fear of infecting with the virus, difficulty in accessing health services during the pandemic, and poor health system services. Several studies reported that individuals self-medicated in order to treat or prevent COVID-19 disease, boost immunity, and lessen anxiety associated with the pandemic. Affected by the policies of COVID-19 pandemic, a number of people also indicated that they self-medicated often based on lockdown, fear of isolation, and fear of stigma.

Of the general causes, the financial factor was the most prevalent, and was reported by half of the studies that described the reasons for self-medication. The remaining usual reasons involved time saving, previous relevant experience, minor illness, distance to health facilities, the suggestion of others, and emergencies. A portion of the research also addressed reasons related to the doctor-patient relationship. In eight studies, participants exhibited distrust of government health institutions or experienced negative experiences with doctors or were unwilling to go to health institutions. In terms of medication knowledge, a few studies indicated that people preferred to self-medicate due to sufficient knowledge of medications, while another study on the other hand revealed that people decided to self-medicate as they were unaware of the adverse effects of the drugs.

Table 4 shows the reasons that drove people to practice self-medication as reported in each study.

**Table 4:** Major reasons for the practice of self-medication.

**Reason for self-medication**		**No. of studies**	**References**
**COVID-19 related reasons**			
Accessibility	Poor access to doctor	10	(29, 33, 42, 46, 48, 55, 56, 59, 64, 68)
	Poor access to health facilities service	9	(29, 53, 55, 59, 68, 73, 79, 81, 82)
	Poor access to medicine in health facilities	2	(81, 82)
	Lockdown	4	(31, 32, 54, 60)
Fear to get COVID-19	Fear of getting contact to virus	12	(29, 31, 33, 39, 42, 55, 56, 59, 67, 68, 79, 82)
	Fear of COVID-19 test	2	(73, 82)
	Fear of being stigmatized or discriminated	2	(81, 82)
	Fear of self-isolation/Quarantine	2	(81, 82)
	Strengthening the immune system	4	(31, 32, 54, 60)
Treatment	To prevent or treat COVID-19 disease	7	(10, 28, 32, 60, 67, 79, 80)
	Treating COVID-19-related anxiety and stress	2	(31, 60)
**General reasons**			
Affordability	High fees/save money on going to the doctor/economic/to save money	17	(28, 29, 33, 37, 41–43, 46, 48, 53, 55, 56, 68, 76, 77, 81, 82)
	The lack of insurance coverage	1	(28)
Personal mobility	Time wastage/to save time/lack of time	12	(29, 33, 39, 42, 46, 48, 53, 68, 76)
Health knowledge	Previous knowledge regarding the problem/previous experience with complaints so that you know how to treat them/previous satisfaction with the medicine	11	(28, 33, 37, 41–43, 52, 53, 55, 68, 76)
	Sufficient knowledge about drugs	2	(53, 76)
	The lack of knowledge about the adverse effects of the drug	1	(28)
Health beliefs	Due to minor problem/disease was not serious/mildness of illness	10	(28, 29, 33, 39, 42, 43, 48, 53, 76, 77)
	It is habit of yours/consume it regularly	4	(10, 56, 59, 67)
	Felt no need to consult a physician	1	(37)
Availability	Too far from the health institution/the location of the health service facility/there is no transportation	8	(28, 33, 41, 55, 68, 76, 81, 82)
	Emergency/to get quick results	6	(37, 41, 52, 53, 81, 82)
	Easy access to medicines (available at home, cheap drugs, over-the-counter sales in pharmacies)	3	(28, 76, 81)
	Lack of effectiveness of doctors' medicine	2	(56, 73)
	Not eligible for treatment	1	(55)
Advice from others	Pharmacist advice/recommended by others, pharmacy, media/Friend, social media, TV, radio program influenced your decision to self-medicate	7	(28, 43, 55, 64, 73, 79, 81)
Negative attitudes toward health services	No faith in government health facilities/I do not trust doctors	6	(28, 37, 55, 59, 68, 73)
	Bad experience with doctor	1	(56)
	Unwillingness to attend a health care facility/use time from a healthcare professional	1	(64)
Personal health management	Treatment acute or chronic illness/reduce the disease symptoms	4	(31, 32, 60, 61)
	Support respondents' activity/to increase physical performance	2	(32, 60)
	Improving general wellbeing	1	(61)
	Psychological assurance	1	(80)

##### 3.3.5. Factors associated with practice of self-medication

Factors associated with self-medication practices were characterized in 39 studies in terms of sociodemographic, anthropological, and pandemic-based data. Table 5 shows how factors like age, gender, education, marital status, occupational status, field of occupation, family income, geography, insurance, socioeconomic status, health status, anxiety, prior self-medication experience, knowledge and attitude toward self-medication, drug use habits, and perception regarding the COVID-19 pandemic correspond with different levels of self-medication habits among research participants.

**Table 5:** Factors associated with self-medication.

**Factor**	**No. of studies**	**References**
**Demographics**		
Gender	20	Female (10, 31, 34, 69, 70, 74) Male (65, 66, 72) Gender (30, 38, 41, 43, 47, 50, 55, 57, 60, 61, 81)
Age	18	18–24 (76); younger (72); adult age (66); 23–28 (39); adult and older adult (>29 years) (65); 25–34 (76); >40 years (69, 79); >35 to 55 (31); >55 (31); more than 60 years old (75); older age (34, 80); The younger and older working mothers (32); Age (41, 44, 47, 50, 57, 67)
Education	18	None/primary (79) Low educational level (elementary and middle school) (69, 70) High school (32) Secondary level (74, 79) Higher diploma, degree or above (31) University level (74) More educated (72) Education (28, 29, 40, 43, 45, 47, 50, 55, 61, 81, 82)
Marital status	10	Single (69, 76) Marital status (30, 38, 47, 48, 50, 57, 80) Widowed/separated (79)
Work/study sector	8	Working in the health sector (74) Occupation (Government employee) (76) Work in the medical field (57) Being pharmacist (80) Health profession (72) Status designation (61) Place of practice (82) Faculty (60) Field of study (43)
Income and expenses	7	Higher income (32, 80) Fewer family expenses (32) Income (43, 47, 52, 75, 82)
Employment	6	Unemployment (69, 75) Profession (44) Currently have a job (67) Occupation (52, 82)
Region	5	(30, 43, 52, 61, 75)
Anxiety	5	Anxiety (34, 38, 60) Psychological distress (72) Being diagnosed with mental illness (69) The use of psychiatric medication (69)
Symptoms	5	The experience of COVID-19 symptoms (60, 78) Cough and flu symptoms (44, 66) Anosmia (66) Dyspnea (66) Dysgeusia (66) Nausea/vomiting (66) Gastroesophageal reflux (66) Dental complaint: Pain (12) Pus and abscess (12)
Grade level (for the student population)	4	(10, 41, 43, 60)
Health status	4	Healthy (9) Self-reported health as good (10) Number of times you fell ill during COVID-19 (44) Number of medications used every day (48) Use of canes/crutches (48)
Family factor	4	Household size: 6 and more (75) Having a health sciences student within the family (65) Having children under 18 in a household (8) Having children (30)
Comorbidity	3	The presence of a comorbidity (44, 65) Diabetes mellitus (80)
Life habits	3	Undertook physical activity (9) Diet (60) Smoking (66)
Insurance	2	Insurance coverage (28) Having private health insurance program (69)
Socioeconomic status	2	Low socioeconomic status (45, 70)
Religion	2	Have a religious affiliation (8, 31)
Work time	1	Worked on day shift (9)
Ethnicity	1	(76)
**Self-medication behavioral aspects**		
History of use	4	History of use TCIM (31, 47) Have previous history of self-medication (52) Number of times taking antibiotics in the past year (72)
Knowledge	4	Poor knowledge about SM (76) Knowledge about SM (60, 72, 81)
Attitude	3	Thinking that chlorine dioxide is not effective (65) Not being informed of the efficacy of chlorine dioxide (65) Your opinion for opting for self-medication during COVID-19 (44) Likelihood of self-medication within next 6 month (29)
Irrational drug use habits	3	Storing medications at home (48, 68) Forget drug use (48) Confuse medication time (48) Having recommended medications to other people (48, 68) Addicted to drugs (30)
Information source	3	Trusted information (ref: Thai govt.) (34): friends/family, doctors online, and foreign countries Having medical information as a source of information about COVID-19 (65) Friends as a source of information (30)
Use of other substances (for self-medication for specific drugs)	3	Medications (65) Plants (65) Use prescription medication (83) Consumption of drugs (69)
Access	1	Took leftover antibiotics (72) Easily acquire antibiotics from friends/family (72) Easily acquire from doctor (72) Asked doctor for antibiotics (72)
Price	1	The perception of self-medication as cheap (79)
**COVID-19 context**		
Consideration of COVID-19	10	COVID-19 pandemic (12) Worries getting infected with COVID-19 (61, 63) Being infected with coronavirus (57), Having any close family member infected with coronavirus (57) Previous COVID-19 testing (30, 80) Those previously infected with COVID-19 (10) Avoid going to the clinic/hospital due to COVID-19 (44, 73) Afraid of the pandemic (50)
Perceived susceptibility	2	Strongly considering COVID-19 to be a dangerous and deadly disease (65) Personal sensitivity (83)
Face masks	1	Taking off face masks in enclosed public places (83) Frequent face-mask use (83)

Gender, age, and education were the most frequently reported factors, which all show contradictory results. Two studies observed that younger age was positively associated with self-medication behavior (39, 72) in contrast to six other studies showing that older age was a favorable correlate for engaging in self-medication (31, 34, 69, 75, 79, 80). Six studies reported that female gender was actively involved in self-medication (10, 31, 34, 69, 70, 74) while three studies observed a higher propensity for male gender (65, 66, 72). With respect to education level, four studies yielded a higher likelihood of self-medication at lower education levels (32, 69, 70, 79), whereas three studies produced results that higher education levels were more likely to self-medicate (31, 72, 74). Working in the medical/health field was revealed to be a favorable predictor of self-medication in 4 studies (57, 72, 74, 80). Two studies reveal a significant relationship between self-medication and insurance coverage (28, 69). Additionally, individuals in two studies with lower socioeconomic position showed a greater propensity for self-medication (45, 70).

There are 10 studies identifying relationships between COVID-19 and self-medication (10, 12, 30, 44, 50, 57, 61, 63, 73, 80). Afraid of the pandemic, fear of being infected, being previously infected, previous COVID-19 testing, and avoiding going to the hospital due to COVID-19 were all factors affecting self-medication. Sources of pharmacological information (30, 34, 65), drug use habits (30, 48, 68), and drug accessibility (72) all have an impact on self-medication.

##### 3.3.6. Sources of medication products and information/recommendation

Twenty-four studies observed sources of information regarding self-medication. Medication information regarding self-treatment came from a diverse range of sources, of which friends/relatives (*n* = 22) and social platforms/networks (*n* = 21) were the most dominant. Healthcare professionals were also a key component of the sources of information about self-medication (*n* = 15), including physicians, pharmacists, nurses and herbalists. Other sources of information included old prescriptions, academic knowledge available from scientific websites/books/research articles, news/TV/radio/advertising, product's brochures, as well as own judgment. In several of the individual research, government agency such as ministry of health help center, cultural influences, and illegal prescribing were also mentioned.

Eighteen researches provided data on the primary sources of products used to treat self-medication. Pharmacies were listed as a purchase channel in all of the publications. Sources of medication acquisition in public setting also included hospitals (*n* = 3), primary health facilities (*n* = 3), private clinics (*n* = 1), stores/shops (*n* = 2), and herbalists (*n* = 2). From the perspective of one's medication habits, numerous investigations discovered that access to medications included relatives and friends (*n* = 7), leftovers at home (*n* = 8), and homemade (*n* = 1). Five research cited online resources, including telemedicine and online pharmacies. Meanwhile, a few studies also identified irregular channels for people to obtain medications such as patent medicine vendor (*n* = 3), hawkers (*n* = 1), and faith-based outlets (*n* = 2).

##### 3.3.7. Knowledge and attitude about self-medication

The majority of the articles that discussed respondents' knowledge of self-medication revealed a moderate or high level of good knowledge. According to a research from Kenya, during the outbreak, knowledge of the dosage, mode of administration, and adverse effects of purchased medicines climbed to 75% (9). The report from Iran shows that only 20% of patients with poor knowledge level about antibiotics (27). In terms of knowledge about adverse effects of self-medication, <½ of the parents (42.7%) reported limited knowledge about side effects in Turkey (46). Of these parents, 103 (62%) believed that the medication caused negative consequences on the gastrointestinal system. In a group of students, Merwid-Lad et al. observed that their knowledge of dietary supplements was rated as moderate or high (60). In four studies assessing self-medication knowledge, the proportion of participants with good awareness was 47.6, 57.4, 58.6, and 96.7%, respectively (52, 68, 76, 81). Amuzie et al. reported that virtually all respondents (97.7%) were aware of self-medication and more than three-quarters (88.4%) correctly defined it (79).

People's attitudes toward self-medication were mixed in the included studies. On the one hand, participants in several research reported that self-medication is unsafe and ineffective as well as not changing symptoms, while having negative long-term impacts on the body, like drug dependence, drug resistance, efficacy reversal and damage to body organs. On the other hand, respondents in some studies maintained a positive attitude toward self-medication. They claimed that they felt better physically or psychologically after self-medication and considered self-medication to be effective, beneficial and safe. Two articles also discussed the relationship between attitudes toward self-medication and COVID-19 pandemic. Onchonga et al. (9) reported that more than half (64.3%) felt that the COVID-19 pandemic necessitated self-medication and would continue self-medication post-COVID-19 pandemic (55.9%). 84.1% felt there was an increased desire for self-medication in the general population as a result of the pandemic. However, others (91.5%) thought that test for side effects should be performed before using Traditional Chinese Medicine in COVID-19 therapy (47).

##### 3.3.8. Adverse drug reactions

A total of 14 articles have described the situation of adverse reactions to self-medication. The proportion of adverse drug reactions associated with self-medication ranged from 4.7 to 36%. Adverse reactions have been reported mainly involving the central nervous system (anxiety, irritability, insomnia, poor concentration, headache, dizziness, fatigue, and sleepiness) and the gastrointestinal system (nausea, vomiting, loss of appetite, diarrhea, bloating, constipation, and stomach pain or heartburn). Other symptoms included drug dependence, dry mouth, allergic reactions, and fungal infections.

#### 3.4. Role of healthcare professionals at the community level

##### 3.4.1. Pharmacist

A total of 12 articles mentioned pharmacists in various contexts. The content primarily covers sources of information about self-medication, suggestions for self-medication, and measures to take after adverse reactions due to self-medication, with one article exploring the role of pharmacist. When investigating the pharmacist's role in self-medication, it was found that respondents asked more frequently about three areas of medication advice, dose use and medication interval, and other areas included combination medication and side effects in self-medication in detail.

There are seven articles that discuss the role of pharmacists in advising on self-medication. The proportion of people who practiced self-medication on the advice of the pharmacist was similar across the three studies, ranging from 17 to 18% (46, 50, 60). In the study by Tobaiqi et al. (55), advice from pharmacists accounted for the third highest reason for self-medication, standing at 27%. In addition, about 19% of the respondents consulted pharmacists about the use of the antibiotics such as dosage, duration, etc. However, the survey by Jiri et al. had a different finding in that pharmacists were infrequent (4.5%) among the sources of advice for self-medication as well as when asked where they obtained their knowledge regarding the hazards of self-medication, just 1.1% obtained it from pharmacists (29). The mean score for the question “it is sufficient for medicines to be prescribed by pharmacists” was moderate (about 4 on a scale of 1–7) as reported by Coman et al. (59). A study carried out on a group of university students in Pakistan found that the advice of the pharmacist was the most popular factor influencing their self-medication. When investigating the pharmacist's role in self-medication, it was found that respondents focused more on three areas of medication advice, dose use and medication interval, and other areas included combination medication and side effects. Furthermore, a subset of the participants received guidance from pharmacists on the side effects of medicines (43).

The function of pharmacists as a provider of self-medication information is inconsistent. For two studies (11, 57), in nearly half, pharmacists were used as a source of information about self-medication, while in the other three studies (31, 53, 73), only a minority of participants did so. Pharmacists can also perform a supporting role following self-medication practices. In a Kenyan study (9), 10.8% of participants decided to consult a pharmacist after experiencing an adverse medication event.

##### 3.4.2. Other healthcare professionals

The role of other healthcare professionals was also described in different studies. Apart from pharmacists, the more commonly mentioned healthcare professionals were physicians including general practitioners and private doctors. Others included psychologist, psychiatrist, chiropractor, massage therapist, dietitian, nurse, and herbalist.

Six articles addressed the role of healthcare professionals in providing advice to individuals on their own use of medication. Participants in two of the studies approached their physicians for advice at similar rates [25% (50); 28.59% (51)]. In the Dehghan et al. study, a higher proportion consulted a physician before using dietary supplements (55%) than before using herbal medicines (33.3%) (30). Two more roles, similar to the previous subsection, were to provide a source of drug information and to deal with adverse events. The study by Mutua et al. said that only 4% received drug information from health practitioners or quacks (73). In the other article, 11.8% of participants indicated that they would take measures to seek a private doctor after an adverse reaction (9).

## 4. Discussion

### 4.1. Major contribution

This review provides a detailed overview of the practice of self-medication in different populations during the pandemic. A large volume of self-medication-related literature was yielded by our search, demonstrating a trend for researchers to spotlight self-medication in medical resource-limited settings like COVID-19 pandemic. To the best of our knowledge, it is the most thorough systematic review of self-medication during a COVID-19 pandemic to date. Self-medication behaviors performed in response to this as yet incompletely clarified COVID-19 disease require a great deal of attention, as do several self-medication situations occurring as a result of changes in the health care resource, environment and services associated with the pandemic. However, previous related systematic reviews limited their scope only to medication use in COVID-19 disease (21, 24, 25). Findings from our results show variations in the prevalence of self-medication reported across different country regions, with differences in the structure of health systems, access to over-the-counter medications, epidemiological policies between countries, as well as the population and purpose of each study influencing trends in self-medication. Moreover, approximately only a quarter of the studies we found addressed the role of health care professionals in guiding self-medication. In light of the potential risks associated with unregulated self-medication, the value of a comprehensive understanding of self-medication practice is even more pronounced.

### 4.2. Self-medication is a common practice during COVID-19 pandemic

The results of this review found that the behavior of higher self-medication during COVID-19 pandemic is of concern. Given the circumstances and structure of health systems, self-medication was a widespread practice in low- and middle-income countries and regions. Meanwhile, it was also observed that there was a higher proportion of self-medication behavior among groups that need more attention in society including general public, older adult and patients. Similar high prevalence rates have been reported in previous studies including pre-pandemic and pandemic periods (2, 3, 19, 21, 25, 84, 85). The heterogeneity caused by separate studies precludes straightforward comparisons. There were, however, a few articles in the available literature that compared self-medication before and after the pandemic. The studies conducted in Iran (12), Kenya (9), and Nigeria (77) all identified a trend of rising self-medication usage during the pandemic relative to the pre-pandemic period. This may relate to the accessibility of health services and the risk of infection in health care settings during the pandemic (12).

### 4.3. Concerns and benefits associated with self-medication during COVID-19 pandemic

Antibiotics, analgesics, vitamins and dietary supplements, herbal medicines were examples of the types of pharmaceuticals that are widely utilized for self-medication during the pandemic. The type of drugs used for self-medication is not without safety concerns. If used improperly, adverse and potentially harmful effects can occur. Paracetamol is primarily used to relieve pain and cold-related symptoms, while vitamin C is commonly utilized to boost the body's immune system (86). This review discovered that paracetamol/acetaminophen, as well as vitamin C, were frequently cited as the medications with the highest percentage of use in the investigations. However, these two drugs will interact in the body, competing for the body's sulfate pool thereby lengthening the paracetamol residence time in the body, which potentially contributes to enhanced toxicity (87). Meanwhile, the review revealed that many research done tend to investigate only the class of pharmaceuticals used for self-medication without mentioning the specific drug ingredients. That may be because for the general public, as non-medical professionals, often rely on the brand name or the indication type of the drug to purchase. However, it is worth noting that antipyretics, cold or flu medications, and compounded medications may carry the same main ingredients which should not be taken together to avoid overdose resulting in hepatotoxicity (88). Excessive intake of vitamin C may cause side effects, which most specifically increase the risk of kidney stones (74).

Among the included studies, antibiotics were the most widely referred to medication (*n* = 35). The inappropriate utilization of antibiotics, encompassing self-medication with residual medications or acquiring them from unreliable sources, represents a significant healthcare concern (89). Individuals may turn to using leftover antibiotics from earlier treatment plans, which creates a hazardous scenario because antibiotics should only be used as directed and for the full period of the specified treatment (90). On the other hand, in many cases, antibiotics are not considered necessary in the treatment of certain symptoms or diseases (91). In the review, symptoms most commonly self-treated by respondents were found to be those related to the common cold and other upper respiratory tract infections (URTIs). Since viruses are primarily to blame for these symptoms, antibiotics shouldn't be used to treat them. Yet the current study showed that the use of antibiotics in such viral illnesses is widespread. This may contribute to the development of antibiotic resistance, thus posing a threat to global health.

The majority of individuals rely on family and friends as sources of drug information, followed by social media and Internet. With the advent of digitalization, people now have a simple access to the internet where they easily research their symptoms and discover what they believe to be the best course of action. However, there are evidence that healthcare misinformation linked to COVID-19 pandemic diffused at alarming rates on social media (92, 93). In addition, researchers have noticed that erroneous information regarding COVID-19 on social media is much more popular and challenging to block from spreading (94). The way that people interpret and respond to false information might vary depending on their environment and culture (95). These practices were risky and may have clinical consequences such as adverse reactions, drug-drug or drug-herb interactions, and antimicrobial resistance.

In this review, it should be emphasized that, other from the fear of infecting the virus, the reasons why people self-medicate for COVID-19 during the pandemic period included inaccessibility and unacceptability. Inaccessibility referred to difficulties in accessing services due to physicians' busy schedules; in terms of acceptability, the health system was perceived to be poorly served during COVID-19. Owing to the immense patient burden during the pandemic, most physicians lack sufficient time to interact well with patients. They tended to concentrate primarily on biomedical elements of body health while ignoring psychological aspects of care. When these conditions are combined with the other bottlenecks experienced throughout the health care delivery process in resource-limited settings, most patients leave the facility dissatisfied, reducing trust and acceptability of health care services (96), which negatively impact health care seeking behavior and lead to more self-medication behavior (97). Hence the government need to be aware of the problems in this area and make timely response solutions in future pandemics.

Before or during the pandemic, financial considerations, time considerations, and minor illnesses were discovered to be common causes of self-medication. Self-medication is a great option to get a more convenient and cheaper treatment for minor illnesses (15). Likewise, this is true for pandemics, where responsible self-medication practices can both prevent the crowding of medical resources and quicker control of the disease's progression. Therefore, more education and awareness measures will be needed to enable the public to better utilize the benefits and reduce the risks of self-medication.

### 4.4. Support is needed for safe practice of self-medication

The high prevalence of self-medication during the COVID-19 pandemic significantly highlighted the importance of maintaining counseling efforts and guidance on medication use, even in situations where health care services are disrupted and/or resources are limited. The WHO suggested that achieving “successful” self-medication in many countries would need increasing people's awareness and education in order to minimize the possible harm that could result from this practice (1). Similarly, the International Pharmaceutical Federation, in tandem with the World Self-Medication Industry (98), and the World Medical Association (99), emphasizes the responsible use of non-prescription medications. Also, self-medication as a key component of self-care. The FIP document (20) indicates that policies should more prominently reflect the benefits of self-care, especially demonstrating how self-care can improve health and wellbeing in complement with formal healthcare systems. Transforming passive patients into proactive participants interested in their own health management, leading to a revolution of the health care system from a disease system to a prevention system, is critical to the advancement of health care. A comprehensive understanding of this global concern will offer clues to the formulation of sound, effective, and efficient public health policies and guidelines to facilitate responsible self-medication and minimize the risks associated with self-medication. This study suggests that supporting responsible self-medication practices necessitates the participation of all key stakeholders and the long-term viability of strategic health promotion and education programs.

Considering the diverse information available to public, authorities need to strengthen pharmaceutical information dissemination as well as safety medication education. Akyol Onder and Ertan (100) suggested that dissemination of factual information would facilitate sensible solutions to the worst public health catastrophe of the century. Receiving trustworthy information from healthcare professionals would be a potent strategy to prevent misinformation and promote responsible self-medication (101). Furthermore, countermeasures can be implemented to prevent the escalation of disinformation by comprehending the patterns of misinformation. Collaboration among fact-checkers, news media, platform companies, and public authorities is necessary to sustain a coordinated effort to address the spread of misinformation about COVID-19 and to assist the general public in understanding and responding to the pandemic (102).

Governmental health departments can facilitate self-care by providing effective, efficient, and inclusive primary care services to the general public, specifically through community pharmacies, high quality health care information, and convenient access to preventive care and complementary care services. Systems will gain over time from the effective distribution of resources among primary and specialized care services. For special populations that require regular monitoring, counseling and medication administration such as those with chronic diseases, and pregnant women, establishing counseling facilities or streamlining the prescription refill process for them would be beneficial and alleviate general anxiety as well as promote community health (36).

### 4.5. Role of the pharmacist

Pharmacists should be positioned as key roles in the public health measures to address self-medication behavior during the pandemic. This is especially the case when concurrent use of prescription medicines and traditional and complementary medicines are involved which gave rise to additional risks to drug safety (103, 104). Our results show that pharmacists were the professionals more frequently mentioned by the public for offering medication information or advice during self-medication. First, public in fact encounter pharmacists more frequently than other healthcare professionals (105). The finding showed that pharmacies were the stakeholders responsible for providing drugs to the self-medicating public in a time of pandemic, which reflects the favorable conditions for pharmacists to make a difference in self-medication. Second, pharmacists are trusted sources of health information in communities, and they promote to generating positive health outcomes by empowering individuals to better care for their own health (20). Furthermore, pharmacists are well-trained to effectively educate patients and provide evidence-based advice on a broad range of topics, including self-care interventions and the use of non-prescription medicines in the treatment of minor ailments (106, 107). Community pharmacists can support curbing the dangers of self-medication by repeatedly communicating (108), monitoring medication-related risks, and identifying populations at risk for substance abuse (109–111). As such, their role in facilitating the provision of safe and effective self-medication practices should be more effectively implemented. From another perspective, the fact that participants in the included studies use old prescriptions and leftover medications at home also points to deficient aspects of prescribing and dispensing which require effective health education and promotion strategies. Strengthening the regulation of dispensing practices while encouraging pharmacists to educate patients about medication use during the consultation process.

For future pandemics, we recommend that pharmacists actively involve their patients in early conversations pertaining to the medications they may use for the prevention and treatment of infectious diseases and instruct them appropriately. The findings of this study may assist them in reflecting on and evaluating the burden of self-medication in society and benefit them in developing strategies to curb the problem. However, barriers to the uptake of pharmacists' engagement in self-medication are multiple (112, 113). In particular, it would need to take into account the recognition of pharmacists in society. Compared to doctors, pharmacists are frequently regarded with less trust (114). To some people, the role of pharmacists remain predominantly traditional, often limited to solely dispensers of medications based on prescriptions (115). Simultaneously, the management patterns and remuneration mechanisms in pharmacies have resulted in a predominant emphasis on the retail activities, rather than prioritizing pharmacist's professional role in providing comprehensive advice and guidance (116). Significant efforts are required if pharmacy is to transform from a “dispenser and seller alone” mentality to a more clinical, patient-centered entity. Insufficient professional self-perception and inadequate training (20) are also among the challenges pharmacists face in supporting self-medication guidance services. Facilitating the ability of pharmacists to effectively support patient self-medication necessitates the cultivation of an enhanced sense of social responsibility and the provision of higher-quality education and training.

Further to this, the responsibility of healthcare providers, including physicians, nurses, dentists, and other healthcare professionals, to increase awareness of the appropriate medication use, both in the context of the pandemic and traditional healthcare settings, needs to be emphasized. Appropriate medication utilization is essential to ensure patient safety, optimize treatment outcomes, and prevent medication errors and adverse reactions (117–119). Healthcare professionals are encouraged to offer patient education using concise and comprehensible language to elucidate the potential advantages and risks linked to medications, while also addressing any concerns or inquiries that patients may present (120). Meanwhile, healthcare providers should emphasize the importance of medication adherence and provide strategies to promote patient compliance (121).

### 4.6. Way forward

Wide disparities in results among studies were discovered in the review's findings, indicating that each setting (region, country), has its own patterns and implications for self-medication, and therefore urged different local health authorities to support research and interventions to lessen the likelihood of unfavorable self-medication outcomes. Further analysis of self-medication trends is also necessary given the quickly shifting COVID-19 situation and the results of widespread immunization. Most studies related to self-medication of COVID-19 disease focused their research scope on the prevention and treatment of COVID-19, while research on self-medication behaviors of people recovering from COVID-19 is also necessary. The prevalence, types of medication, contributing factors, and adverse effects of self-medication during the pandemic have been the subject of several research; nevertheless, little is known about poor medication habits or medication misconceptions like drug combination, overdose, or repetitive medication usage. Consequently, more qualitative, comprehensive, and comparative studies will provide a richer and deeper comprehension of the phenomenon of self-medication and thus better guide future practice. Meanwhile, during the pandemic, self-medication has become an essential health policy, yet current studies have mostly concentrated on the adverse effects of self-medication, so there is a call for more research to explore the beneficial aspects of self-medication and the implementation of self-medication health policies.

The results of our review found that only one in five studies mentioned pharmacists as a source of information or advice on self-medication. Considering the importance of enhancing responsible self-medication practices through the mentorship of health practitioners, particularly pharmacists, and the research gaps in this area, the findings of this study will drive future contextual and insightful research. Greater research is warranted to explore the potential value of pharmacists in guiding people to self-medicate and how government can support pharmacists in developing such role to meet the needs.

### 4.7. Strengths and limitations

The results of this review should be viewed with caution, as the key messages of each study have been carefully reviewed and provide important guidance for contemporary medical practice. The main limitation of this systematic review was the heterogeneity of definitions for self-medication in the examined studies, which made it impossible to do meta-analyses for all of the studies. Also of note, there have been variations in the quality of the included studies, the operational definitions of medicine use, study design, data collection tools, sample selection, sample size, and measurement time frame. We realize that the studies were not randomly distributed across regions, most of which were from Asia and Africa, and that more studies may have been conducted in regions where high self-medication was suspected. Other limitations of this review pertain to cross-sectional research design, variable recall period, and inherent constraints of self-reporting methodology.

## 5. Conclusion

During the COVID-19 pandemic, self-medication practices were widespread and varied across countries and populations. Self-medication has emerged as an important component of health care, but also as a huge global challenge. Self-medication practices may reduce the burden on health care resources especially in COVID-19 scenarios, but may also have potentially harmful and dangerous effects. Therefore, the engagement of healthcare administrators and policy makers and the implementation of health education programs are essential to regulate and monitor appropriate self-medication practices. The expertise and favorable conditions of pharmacists make them positioned as key roles in public health interventions for self-medication. As the fight against COVID-19 continued, more research is needed in the future to explore aspects of self-medication policy implementation and the potential value of pharmacists in self-medication behaviors.

## Data availability statement

The original contributions presented in the study are included in the article/Supplementary material, further inquiries can be directed to the corresponding author.

## Author contributions

YZ and JL conceived of the design, methodology for this review, developed the review protocol, searched the literature with input, analyzed and interpreted the results, prepared the tables and figures, and drafted the manuscript. PT assisted in data analysis, interpreted the results, and reviewed the manuscript. HH supported data analysis, interpreted the results, and critically reviewed the manuscript. CU conceptualized and organized the study, confirmed and interpreted results, and critically reviewed and revised the manuscript. All authors contributed to the article and approved the submitted version.
