# Supplementary material for: A systematic review of self-medication practice during the COVID-19 pandemic: implications for pharmacy practice in supporting public health measures
Source: Front Public Health. 2023 Jun 15;11:1184882. doi: 10.3389/fpubh.2023.1184882 (PMC10310324; doi:10.3389/fpubh.2023.1184882)
Supplement: Supplementary file 2 [file Table_2.DOCX]

Supplementary Material

A systematic review of self-medication practice during the COVID-19 pandemic: implications for pharmacy practice in supporting public health measures

Yu Zheng ^1,†^, Jiayu Liu ^1,†^, Pou Kuan Tang ^1^, Hao Hu^1,2^, Carolina Oi Lam Ung^1,2,*^

^1^ State Key Laboratory of Quality Research in Chinese Medicine, Institute of Chinese Medical Sciences, University of Macau, Macao, China

^2^ Department of Public Health and Medicinal Administration, Faculty of Health Sciences, University of Macau, Macao, China

*** Correspondence:**Carolina Oi Lam Ung
carolinaung@um.edu.mo

**^†^** These authors contributed equally to this work and share first authorship

# Supplementary Tables

**Search strategies**

**Database:** PubMed

**Date:** 2022-07-22

**No of results:** 1198

| **ID** | **Searches** | **Results** |
| --- | --- | --- |
| #1 | "covid 19"[MeSH Terms] OR "covid"[Title/Abstract] OR "severe acute respiratory syndrome coronavirus 2"[Title/Abstract] OR "2019nCoV"[Title/Abstract] OR "novel coronaviru*"[Title/Abstract] OR "sars coronavirus 2"[Title/Abstract] OR "SARS-CoV-2"[Title/Abstract] OR "sars2"[Title/Abstract] OR "new coronaviru*"[Title/Abstract] OR "coronavirus disease 2019"[Title/Abstract] | 274246 |
| #2 | "self-medication"[MeSH Terms] OR "self-care"[MeSH Terms] OR "self management"[MeSH Terms] OR "self-medication"[Title/Abstract] OR "self-care"[Title/Abstract] OR "self treatment"[Title/Abstract] OR "self management"[Title/Abstract] OR "self intervention"[Title/Abstract] OR "nonprescription drugs"[MeSH Terms] OR "nonprescription drugs"[Title/Abstract] OR "over the counter drugs"[Title/Abstract] OR "otc drugs"[Title/Abstract] OR "drug utilization"[Title/Abstract] OR "medication utilization"[Title/Abstract] OR "self remedy"[Title/Abstract] OR "self-prescription"[Title/Abstract] OR "self-administration"[Title/Abstract] | 106855 |
| #3 | #1 AND #2 | 1495 |
| #4 | (#1 AND #2) NOT (#1 AND #2 AND (booksdocs[Filter] OR clinicaltrialprotocol[Filter] OR comment[Filter] OR editorial[Filter] OR letter[Filter] OR meta-analysis[Filter] OR news[Filter] OR newspaperarticle[Filter] OR review[Filter] OR systematicreview[Filter])) | 1198 |

**Database:** Web of Science

**Date:** 2022-07-22

**No of results:** 1016

| **ID** | **Searches** | **Results** |
| --- | --- | --- |
| #1 | TS=("covid 19" OR "severe acute respiratory syndrome coronavirus 2" OR "2019nCoV" OR "novel coronaviru*" OR "sars coronavirus 2" OR "SARS-CoV-2" OR "sars2" OR "new coronaviru*" OR "coronavirus disease 2019") | 319307 |
| #2 | TS=("self-medication" OR "self-care" OR "self management" OR "self treatment" OR "self intervention" OR "nonprescription drugs" OR "over the counter drugs" OR "otc drugs" OR "drug utilization" OR "medication utilization" OR "self remedy" OR "self-prescription" OR "self-administration") | 81798 |
| #3 | #1 AND #2 | 1268 |
| #4 | (#1 AND #2) NOT (DT==("REVIEW" OR "EDITORIAL MATERIAL" OR "MEETING ABSTRACT" OR "LETTER" OR "BOOK CHAPTER")) | 1016 |

**Database:** EBSCOhost

**Date:** 2022-07-22

**No of results:** 742

| **ID** | **Search** | **Results** |
| --- | --- | --- |
| S1 | TI "covid 19" OR "severe acute respiratory syndrome coronavirus 2" OR "2019ncov" OR "novel coronaviru*" OR "sars coronavirus 2" OR "sars-cov-2" OR "sars2" OR "new coronaviru*" OR "coronavirus disease 2019" | 144823 |
| S2 | AB "covid 19" OR "severe acute respiratory syndrome coronavirus 2" OR "2019ncov" OR "novel coronaviru*" OR "sars coronavirus 2" OR "sars-cov-2" OR "sars2" OR "new coronaviru*" OR "coronavirus disease 2019" | 305749 |
| S3 | S1 OR S2 | 345996 |
| S4 | TI "self-medication" OR "self-care" OR "self management" OR "self treatment" OR "self intervention" OR "nonprescription drugs" OR "over the counter drugs" OR "otc drugs" OR "drug utilization" OR "medication utilization" OR "self remedy" OR "self-prescription" OR "self-administration" | 75106 |
| S5 | AB "self-medication" OR "self-care" OR "self management" OR "self treatment" OR "self intervention" OR "nonprescription drugs" OR "over the counter drugs" OR "otc drugs" OR "drug utilization" OR "medication utilization" OR "self remedy" OR "self-prescription" OR "self-administration" | 215048 |
| S6 | S4 OR S5 | 233106 |
| S7 | S3 AND S6 | 742 |

**Database:** Scopus

**Date:** 2022-07-22

**No of results:** 1596

| **ID** | **Search** | **Results** |
| --- | --- | --- |
| #1 | TITLE-ABS-KEY ( "covid 19" OR "severe acute respiratory syndrome coronavirus 2" OR "2019ncov" OR "novel coronaviru*" OR "sars coronavirus 2" OR "sars-cov-2" OR "sars2" OR "new coronaviru*" OR "coronavirus disease 2019" ) | 369820 |
| #2 | TITLE-ABS-KEY ( "self-medication" OR "self-care" OR "self management" OR "self treatment" OR "self intervention" OR "nonprescription drugs" OR "over the counter drugs" OR "otc drugs" OR "drug utilization" OR "medication utilization" OR "self remedy" OR "self-prescription" OR "self-administration" ) | 164769 |
| #3 | #1 AND #2 | 2335 |
| #4 | #3 AND NOT ( DOCTYPE ( "re" OR "le" OR "no" OR "ed" OR "ch" OR "cr" ) | 1596 |

**Google Scholar**

Go to <https://scholar.google.com/>

**Search the following:** self-medication medicine use covid

Review the first 200 results
